# Cross-Cultural Variation in BMI, Sedentary Behavior, and Physical Activity in International School Girls Residing in Saudi Arabia

**DOI:** 10.3390/ijerph17062057

**Published:** 2020-03-20

**Authors:** Adel A. Alhusaini, Ganeswara Rao Melam, Syamala Buragadda

**Affiliations:** 1 Department of Rehabilitation Sciences, College of Applied Medical Sciences, King Saud University, Riyadh 11433, Saudi Arabia; aaalhusaini@ksu.edu.sa; 2 Department of Rehabilitation Sciences, College of Applied Medical Sciences, King Saud University, Riyadh 11495, Saudi Arabia; sbadari@ksu.edu.sa

**Keywords:** body mass index, cross cultural, expatriate school children, physical inactivity, school girls

## Abstract

Background: The current study was done to assess the cross-cultural difference in physical activity and sedentary behavior among girls from culturally, environmentally, and geographically diverse countries residing in Saudi Arabia. Methods: This was a comparative cross-sectional study conducted among expatriate girls (N = 275), aged 9–16 years. Participants were from India (n = 65), Pakistan (n = 88), Egypt (n = 50), Sudan (n = 49), and other nationals (n = 23). They were randomly selected from different schools in Riyadh and their Body Mass Index (BMI) and screen time was assessed. Physical activity (PA) and leisure-time activity was assessed using Physical Activity Questionnaire for Older Children (PAQ-C) and the Godin-Shephard Leisure-Time Exercise Questionnaire (GSLTPAQ) respectively. Results: Out of 275, 65.8% were active and 34.2% were insufficiently active as per the GSLTPAQ, and half of them were moderately active and only 22.2% were extremely active as per PAQ-C. No statistical significant differences in their BMI status, screen time, or the levels of PA among expatriate girls. Conclusions: This study shows that the expatriate female school children in Saudi Arabia demonstrated a similar pattern in their BMI, sedentary time spent, and PA levels.

## 1. Introduction

Overweight or obesity, and physical inactivity are considered important risk factors of non-communicable diseases (NCDs) and considered as one of the leading factors of global mortality [1]. Worldwide, physical inactivity is the cause of 6% of deaths each year and it is the fourth most frequent cause of death in adults [2]. Childhood obesity has become a global epidemic in not only developed nations, but in developing countries as well. This condition is likely to continue to adulthood and is significantly associated with many comorbidities [3]. It is estimated that 23.1% of Saudi children within the age group of 5–18 years are overweight and 9.3% obese [4], and this rate of obesity prevalence is a matter of concern in the Kingdom of Saudi Arabia. The current tendency is approximately double to the prevalence reported over the past two decades [5,6]. 

Rapid urbanization and economic growth in recent years have led to changes in the standard of living, leading to physical inactivity, sedentary behavior, and unhealthy habits in Saudi children and adolescents, consequently contributing to greater incidence of overweight and obesity [7,8,9]. Expatriates from countries like India, Pakistan, Egypt, and Sudan consider Saudi Arabia as their second home and currently 9.2 million of them work in private and government institutions. The standard of living is a reflection of the dominant culture in any country, which can affect expatriates in order to boost their social participation [10]. In the era of rapid globalization, expatriate children and adolescents are no exception to changes in the trend of lifestyle over a period of time leading to acculturation. 

The body composition of an individual depends on familial, genetic, metabolic, demographical, cultural, and socioeconomic factors which often interplay with each other. Interaction of the genetic traits with culturally patterned behaviors and beliefs is likely to be the etiology underlying obesity [11]. The World Health Organization emphasized an action plan to prevent and manage risk factors related to NCDs [12,13]. Some of these factors are non-modifiable and others are preventable and modifiable [14]. Physical activity (PA) and sedentary behavior (SB) were considered as one of the major independent, modifiable risk factors with a significant association with obesity. Although they are two different behavioral domains representing two separate constructs, not merely functional opposites, they have an independent association with the various risk factors of non-communicable diseases [15,16].The two behavioral tendencies such as low physical activity and more sedentary lifestyle can have a significant influence on their inherent sociocultural factors. Recent studies showed that childhood lifestyle behavior may be influenced by socio-cultural and environmental factors [17,18]. Therefore, the patterns of energy expenditure may vary according to their diverse experiences of lifestyle, especially in developed countries when compared to developing countries. Therefore, an understanding of cross-cultural differences in obesity, physical inactivity, and sedentary behavior may be beneficial to understand how different cultures and environments influence lifestyle behaviors of school children. Though reliable and latest evidence on PA and SB of different countries will be of great interest for researchers and policy makers, comparing across a racially varied population is challenging. This may help them to implement policies and programs for early identification and plan possible intervention strategies to reduce mortality and morbidity due to NCDs [19,20]. 

The aim of the present study was to assess the cross-cultural variations related to BMI, the level of PA and SB in expatriate school girls residing in the Kingdom of Saudi Arabia. Cross-cultural comparison may help to understand the PA and SB among expatriate children residing in the Kingdom.

## 2. Materials and Methods

The study was conducted in Riyadh, from August 2015–2016, among female school children age ranging from 10 to 16 years. The final sample included was 275 after the removal of incomplete data for the analysis. This study was approved by the Institutional Review Board at King Saud University, ethical approval number CAMS 35–34/35. School authorities and the participants’ parents signed a written informed consent. 

## 3. Anthropometric Measurements

Demographic characteristics such as child’s height and weight were measured by the researcher as per standard protocol. Participants’ weight was measured using a comprehensive body composition monitor (BF 511; Omron). The measure is estimated to the nearest 100 grams and is considered the final measurement. Height was calculated using a calibrated scale and the measure is estimated to the nearest centimeter and is taken as the final measurement. Participants were instructed to wear minimal clothing with shoes off in an erect standing posture. These values were used to calculate body mass index (BMI) (kg/m^2^) and subsequently classified using the Centers for Disease Control and Prevention’s (CDC) BMI charts [21].

## 4. Assessment of Lifestyle Factors

### 4.1. Sedentary Behavior (SB)

The average number of hours in sitting, watching television, playing video games is considered sedentary behavior and results in low physical activity and less energy expenditure (≤1.5 metabolic equivalents (METs)) [22]. A subjective questionnaire was used to assess SB which was categorized on the daily average time spent on: (1) TV viewing; (2) use of computers for playing video games, web browsing; (3) homework; and the (4) overall sedentary time. We considered two hours maximum screen time as the cut off score based on the American Academy of Pediatrics (AAP) recommendations [23].

### 4.2. Physical Activity Measures

Physical Activity Questionnaire for Children (PAQ-C): This is a subjective measure of PA and is widely used in children [24,25,26,27]. The SB score ranges from 1 to 5: 1 is considered very sedentary, while 5 means very active [26,27]. The level of activity was categorized according to the mean scale of the nine items as low (≤2), moderate (>2 and ≤3), and vigorous activity (>3) [28]. The scale has a total of nine items; each scored on a 5 point Likert scale, and can be managed in the school setting. It is both a valid and reliable tool to measure activity in children.

### 4.3. Godin-Shephard Leisure-Time Exercise Questionnaire (GSLTPAQ)

Leisure-time physical activity can be subjectively assessed using GSLTPAQ [29,30], and is a reliable and valid tool for children [31]. Total leisure time physical activity is expressed as metabolic equivalents (MET) for each level of intensity and is calculated as; (strenuous × 9 METS) + (moderate × 5 METS) + (light × 3 METS). The intensity of PA was calculated as units and the following categories were adopted: ≥24 units relate to ≥14 kcal/kg/week; 14 to 23 units considered 7 and 13.9 kcal/kg/week; less than 14 units is < 7 kcal/kg/week. Using only moderate and strenuous scores, those with a leisure score index ≥24 were classified as active; those with a score ≤23 were classified as insufficiently active with respect to American and Canadian PA guidelines [32,33,34]. 

## 5. Data Analysis

Data were analyzed using SPSS (Statistical Package for the Social Sciences) for Windows, version 22 (SPSS, Chicago, IL, USA). Mean and standard deviation (SD) were calculated for descriptive data. Chi-square (χ^2^) analysis was used to examine the variation in the level of physical activities categorized by GSLTPAQ and PAQ-C. The group differences in PA and SB were analyzed using analysis of variance (ANOVA). 

## 6. Results

Table 1 shows the descriptive characteristics such as nationality, age, height, and BMI of expatriate girls (n = 275) aged 9–16 years. The BMI was categorized based on BMI-for-age percentile on a CDC BMI-for-age growth chart. Of the total participants, irrespective of their nationality, 19.6% were overweight and 18.2% obese. No significant difference in BMI was observed between the groups using ANOVA; *p* = 0.11(Table 1). 

Table 2 shows the difference in screen time (TV+PC) and total sedentary time spent, number of sedentary hours spent per day including the time spent doing homework. No significant difference was found in screen time among the girls using ANOVA (*p* ≥ 0.05).

**Figure 1:**
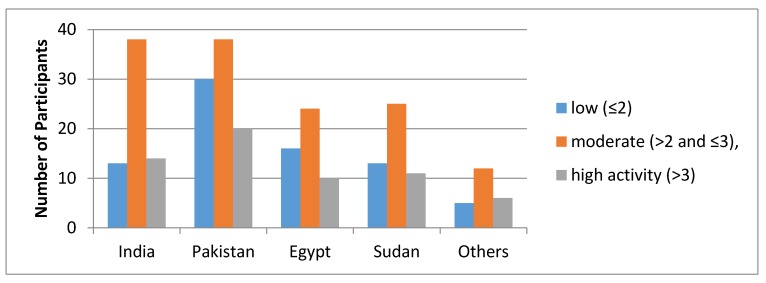
Level of physical activity categorized using Physical Activity Questionnaire for Older Children (PAQ-C).

**Figure 2:**
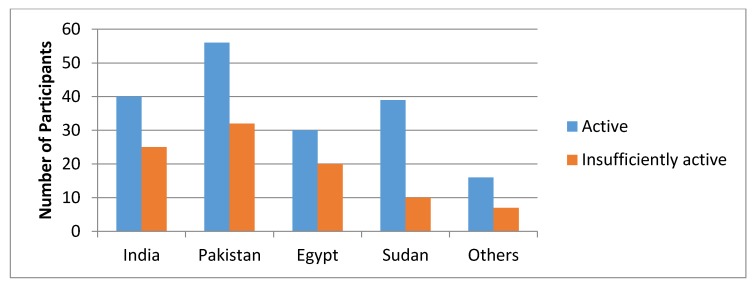
Level of physical activity categorized using Godin-Shephard Leisure-Time Exercise Questionnaire (GSLTPAQ).

The level of physical activity categorized by both PAQ-C and GSLTPAQ is shown in Figure 1 and Figure 2, respectively.

As per PAQ-C, 28.0% of participants had low, 49.8% moderate, and 22.2% had high activity. Similarly, out of 275, 65.8% were classified as active and 34.2% were insufficiently active as per the GSLTPAQ categorical grading. Difference in PA levels (low, moderate, and high) between the groups was assessed using Chi-square test. There was no significance difference in both PAQ-C variables (X^2^ = 5.414, *p* = 0.71) as well as in leisure time activity (*X*^2^ = 5.743, *p* = 0.21) (Table 3). 

No significant difference in the level of physical activity among girls of different countries was observed. There was no significant difference in PAQ-C scores among girls from different countries (F-Ratio = 0.558, *p* = 0.693) (Table 4). Similarly, no significant difference reported with regard to GSLTPAQ scores as determined by one-way ANOVA F-Ratio = 1.592, *p* = 0.177 (Table 5).

## 7. Discussion

It is critical to understand the differences in physical activity as it provides insight into the current and future health problems in both children and adults. The present cross-sectional comparative study assessed the BMI, activity levels, and the amount of sedentary behavior among expatriate female school children in Saudi Arabia. No much literature is available in regard to cross-cultural variation in BMI, PA, and SB characteristics of expatriate schoolchildren. To our knowledge, this is the first ever study done to compare body indices, PA, and SB among children from different countries residing in Saudi Arabia. 

This study provided data about BMI, SB, and PA characteristics with different cultural backgrounds, aged 9–16 years. Sedentary behaviors adopted in childhood may carry on to adulthood. Although statistical difference was not reported with respect to BMI, SB, and PA characteristics, some interesting findings were noted. The overweight and obese children are reported based on the BMI-for-age growth chart and no significant variation based on their nationality. Most of our participants exceeded the recommended international guidelines of screen time, more than 120 minutes daily screen time. The mean sedentary time spent watching TV and computer (4.0 ± 4.76) and overall sedentary time (6.9 ± 3.24) was higher in Egyptian children than their counterparts from other countries. However, there was no statistically significant difference between the groups (*p* = 0.09). The study results are similar to a recent study by Ahmed et al. (2020) which showed that screen time is higher in Saudi boys and expatriate girls [35]

Physical inactivity is associated with many chronic conditions including obesity and is considered pandemic. Increasing physical activity helps to treat obesity [36]. Many subjective and objective measures of PA are available to measure the activity level of a person. In the present study, PAQ-C and GSLTPAQ were used to assess PA. With regard to leisure time PA, 34.2% of children are reported as insufficiently active and significant proportion of children are classified moderately active as per nonleisure-time PAQ-C guidelines. The reduced amounts of either leisure or non-leisure activity in expatriate children residing in Saudi Arabia can be attributed to culture and environmental aspects. Not only environmental factors, but also social and biological factors can be attributed to reduced physical activity and obesity [37]. 

A recent study conducted to determine the factors related to both leisure and non-leisure time activity among Saudi children aged between 14 and 19 years found that female older children were less active than older boys, more specifically in leisure-time activities [38]. Some authors believe that lack of encouragement from parents and the hot climatic conditions are the main reasons for low PA among girls in the Kingdom [18]. Similarly, another study examined the multicultural variation in PA between Saudi and British children. They believed different sociocultural aspects influence the incidence of obesity and PA [19]. Another recent study found that expatriate children were physically active compared to Saudi children of the same age and gender. They also found a negative relationship between body composition and physical activity and a positive relationship between screen time and BMI [35]. Similarly, we also found the same pattern of PA, but we were unique in portraying the results of different sociocultural background people living in the same environmental context. 

It is noteworthy that low physical activity was prevalent in Arab children [39,40] and expatriate children are no exception in this regard. The prevailing culture of society can have a profound influence on the lifestyle factors of the people. In the present scenario of global immigration, many skilled workers from different nations come to Saudi Arabia for the sake of livelihood. The traditional lifestyle of Saudi Arabia provides Saudi women with a few opportunities to avail recreation facilities and to exercise in public. In addition, the different lifestyle factors of expatriate children from different sociocultural backgrounds can have a conflict with the traditional lifestyle of Saudi Arabia. Therefore, we focused on female school children rather than males and this is considered the main strength of our study. Besides the strength of the study, a few limitations need to be considered. The main limitation would be the nature of the sample as the participants were recruited only from the capital city, Riyadh. Therefore, our study results must be interpreted with caution. Future studies should consider different age groups of both boys and girls, other lifestyle factors, dietary behaviors, and different regions of the Kingdom which may have impact on the BMI, PA, and SB. Another limitation was the subjective assessment of PA. Standardized tools such as accelerometers may be used in future studies to provide a more objective amount of physical activity in children.

## 8. Conclusions

Obesity among expatriate children residing in Saudi Arabia poses a serious health concern. This study helped to provide the cross-cultural variation in the level of both leisure and non-leisure activity among expatriate female school children in Riyadh. We concluded that the sociocultural aspects of different nationalities have no significant variation in their BMI, PA, and SB characteristics, and this lack of change might be attributed to the exposure of the same environmental and lifestyle factors, although a certain variation in their dietary habits does exists.

## Figures and Tables

**Table 1:** Descriptive analysis of the participants.

Nationality N (%)	Age (Years)	BMI (kg/m^2^)	Underweight (N = 18; 6.5%)	Normal (N = 153; 55.6%)	Overweight (N = 54; 19.6%)	Obese (N = 50; 18.2%)
India 65 (23)	11.1 ± 1.32	19.7 ± 4.07	7 (2.5%)	33 (12.0%)	15 (5.5%)	10 (3.6%)
Pakistan 88 (32)	13.0 ± 1.64	21.2 ± 4.83	4 (1.5%)	57 (20.7%)	16 (5.8%)	11 (4.0%)
Egypt 50 (18.2)	12.5 ± 1.56	21.9 ± 4.22	1 (0.4%)	26 (9.5%)	13 (4.7%)	10 (3.6%)
Sudan 49 (17.8)	12.2 ± 1.43	21.3 ± 5.27	4 (1.5%)	25 (9.1%)	6 (2.2%)	14 (5.1%)
Others 23 (8.4)	12.2 ± 1.40	20.5 ± 4.52	2 (0.7%)	12 (4.4%)	4 (1.5%)	5 (1.8%)
*p* value		0.11 *	0.22 *			

N—number of participants, %—percentage, BMI—body mass index; * not significant at *p* ≤ 0.05.

**Table 2:** Screen time and total sedentary time.

Nationality	Screen Time (TV+PC) (Hours per Day)	Total Sedentary Time (Hours per Day)	F-Ratio	Significance Level (*p* ≤ 0.05)
India	2.6 ± 1.25	5.5 ± 2.30	1.97	0.09 *
Pakistan	3.7 ± 2.50	6.6 ± 2.79		
Egypt	4.0 ± 4.76	6.9 ± 3.24		
Sudan	3.8 ± 3.10	6.7 ± 3.20		
Others	3.6 ± 2.35	6.7 ± 3.04		

ANOVA analysis, TV—television, and PC—personal computer; * not significant at *p* ≤ 0.05.

**Table 3:** Physical activity levels categorized by Physical Activity Questionnaire for Older Children (PAQ-C) and Godin-Shephard Leisure-Time Exercise Questionnaire (GSLTPAQ).

N	PAQ-C Categorical			GSLTPAQ Categorical	
	Low (≤2)	Moderate (>2 and ≤3)	High Activity (>3)	Active N (%)	Insufficiently Active N (%)
India	13 (4.7%)	38 (13.8%)	14 (5.1%)	40 (14.5%)	25 (9.1%)
Pakistan	30 (10.9%)	38 (13.8%)	20 (7.3%)	56 (20.4%)	32 (11.6%)
Egypt	16 (5.8%)	24 (8.7%)	10 (3.6%)	30 (10.9%)	20 (7.3%)
Sudan	13 (4.7%)	25 (9.1%)	11 (4.0%)	39 (14.2%)	10 (3.6%)
Others	5 (1.8%)	12 (4.4%)	6 (2.2%)	16 (5.8%)	7 (2.5%)
Total	77 (28.0%)	137 (49.8%)	61 (22.2%)	181 (65.8%)	94 (34.2%)
Chi-Square (x^2^)	5.414			5.743	
*p* value	0.71 *			0.21 *	

* Not significant at *p* ≤ 0.05.

**Table 4:** Group differences in PAQ-C.

Source of Variation	Sum of Squares	Degrees of Freedom (DF)	Mean Square	F-Ratio	Significance Level
Between groups (influence factor)	1.1711	4	0.2928	0.558	*p* = 0.693
Within groups (other fluctuations)	141.6234	270	0.5245		
Total	142.7945	274			

**Table 5:** Group differences in leisure-time exercise (GSLTPAQ).

Source of Variation	Sum of Squares	DF	Mean Square	F-Ratio	Significance Level
Between groups (influence factor)	11559.2169	4	2889.8042	1.592	*p* = 0.177
Within groups (other fluctuations)	489,969.6922	270	1814.7026		
Total	501,528.9091	274			

## Abbreviations

BMI	body mass index
CDC	Centers for Disease Control and Prevention
GSLTPAQ	Godin-Shephard Leisure-Time Exercise Questionnaire
MET	metabolic equivalents
NCD	non communicable diseases
PA	physical activity
PAQ-C	Physical Activity Questionnaire for Older Children
SB	sedentary behavior
